# *Chlamydia pneumoniae*-Mediated Inflammation in Atherosclerosis: A Meta-Analysis

**DOI:** 10.1155/2015/378658

**Published:** 2015-08-09

**Authors:** Simone Filardo, Marisa Di Pietro, Alessio Farcomeni, Giovanna Schiavoni, Rosa Sessa

**Affiliations:** ^1^Section of Microbiology, Department of Public Health and Infectious Diseases, “Sapienza” University, Rome, Italy; ^2^Section of Statistic, Department of Public Health and Infectious Diseases, “Sapienza” University, Rome, Italy

## Abstract

Several studies have attempted to relate the *C. pneumoniae*-mediated inflammatory state with atherosclerotic cardiovascular diseases, providing inconsistent results. Therefore, we performed a meta-analysis to clarify whether *C. pneumoniae* may contribute to the pathogenesis of atherosclerosis by enhancing inflammation. 12 case-control, 6 cross-sectional, and 7 prospective studies with a total of 10,176 patients have been included in this meta-analysis. Odds Ratio (OR) with a 95% confidence interval was used to assess the seroprevalence of *C. pneumoniae* and differences between levels of inflammatory markers were assessed by standard mean differences. Publication bias was performed to ensure the statistical power. hsCRP, fibrinogen, interleukin- (IL-) 6, TNF-*α*, and IFN-*γ* showed a significant increase in patients with atherosclerosis compared to healthy controls (*P* < 0.05), along with a higher seroprevalence of *C. pneumoniae* (OR of 3.11, 95% CI: 2.88–3.36, *P* < 0.001). More interestingly, hsCRP, IL-6, and fibrinogen levels were significantly higher in *C. pneumoniae* IgA seropositive compared to seronegative atherosclerotic patients (*P* < 0.0001). In conclusion, the present meta-analysis suggests that *C. pneumoniae* infection may contribute to atherosclerotic cardiovascular diseases by enhancing the inflammatory state, and, in particular, seropositivity to *C. pneumoniae* IgA, together with hsCRP, fibrinogen, and IL-6, may be predictive of atherosclerotic cardiovascular risk.

## 1. Introduction

Atherosclerosis, a chronic inflammatory disease of multifactorial aetiology, typically begins with endothelial dysfunction followed by low density lipoprotein (LDL) infiltration of the arterial intima, mononuclear cell recruitment into vascular wall, and the differentiation of macrophages in foam cells [1, 2]. Macrophages and foam cells, within the evolving atherosclerotic lesion, secrete various proinflammatory cytokines, such as interleukin- (IL-) 6, interferon- (IFN-) *γ*, tumor necrosis factor- (TNF-) *α*, and chemokines, including intercellular adhesion molecule- (ICAM-) 1 and vascular cell adhesion molecule- (VCAM-) 1. In response to proinflammatory cytokines, endothelial cells express high levels of leukocyte adhesion molecules on their surface, leading to further mononuclear cell recruitment and, hence, to a chronic inflammatory state [3, 4]. As a result, the formation, progression, and destabilisation of atherosclerotic plaque occur, leading to cardiovascular diseases, a major public health problem in developed countries, accounting for one-third of all deaths worldwide [5].

In the last decades, several infectious agents have been related to the pathogenesis of atherosclerotic cardiovascular diseases, and current opinion is that the most implicated pathogen is* Chlamydia pneumoniae*, an obligate intracellular microorganism, known as being responsible for respiratory tract infections [6, 7].

The association between* C. pneumoniae* and atherosclerotic cardiovascular diseases has been well documented by seroepidemiological studies [8–12], direct detection of microorganism within atherosclerotic plaque [13–17], and in vivo studies showing an atherosclerotic lesion exacerbation following* C. pneumoniae* inoculation of hyperlipidemic animal models [18–23].

*C. pneumoniae* is presumed to play a role in atherosclerotic cardiovascular diseases for its ability to systematically disseminate from the lung through peripheral blood mononuclear cells and to localise in extrapulmonary tissues, such as the vascular wall [24–28]. Once being inside the vascular tissue,* C. pneumoniae* has been shown to act directly on the cells involved in atherosclerotic process, contributing to endothelial dysfunction, foam cell formation, vascular smooth muscle cell (VSMC) proliferation and migration, and platelet aggregation [29–33]. Indeed,* C. pneumoniae* is able to multiply within macrophages, platelets, endothelial cells, and VSMCs and to induce the elicitation of proinflammatory cytokines, such as IL-6, IFN-*γ*, and TNF-*α*, and adhesion molecules, such as ICAM-1 and VCAM-1, as well as reactive oxygen species, thus contributing to the chronic inflammatory state responsible for the initiation, progression, and destabilisation of atherosclerotic plaque [34–40].

Specifically,* C. pneumoniae* persistent form seems to be responsible for chronic infection and, hence, for the inflammatory process underlying atherosclerosis, since it is able to endure for a long time inside host cells [41, 42].

In addition to the direct effect on vascular cells, previously described,* C. pneumoniae* has also shown to contribute to the systemic inflammation involved in the pathogenesis of atherosclerotic cardiovascular diseases, as evidenced by high levels of IL-6 and high sensitivity c-reactive protein (hsCRP) [7, 43].

Given the central role of inflammation in the atherosclerotic process and the inflammatory effects of* C. pneumoniae*, several studies have attempted to relate the* C. pneumoniae*-mediated inflammatory state with atherosclerotic cardiovascular diseases, providing inconsistent results.

We performed, therefore, a meta-analysis to clarify whether* C. pneumoniae* may contribute to the pathogenesis of atherosclerotic cardiovascular diseases by the means of enhanced inflammatory state.

## 2. Materials and Methods

### 2.1. Search Strategy

We performed a systematic search of all articles in journals indexed on the electronic databases PubMed and Scopus up to December 2014. The search terms used were “chlamydia pneumoniae” or “chlamydophila pneumoniae,” “inflammatory markers” or “inflammation,” “atherosclerosis,” and “cardiovascular disease.” The reference lists of reviews and retrieved articles were hand-searched simultaneously. When more than one of the same patient population was included in several publications, only the most recent or complete study was included in this meta-analysis.

### 2.2. Inclusion and Exclusion Criteria

The inclusion criteria in this meta-analysis were as follows: (i) studies comparing atherosclerotic patients with healthy subjects (control group) in relation to the seropositivity to* C. pneumoniae* IgG or IgA, (ii) studies comparing atherosclerotic patients in relation to the seropositivity to* C. pneumoniae* IgG or IgA, and (iii) studies analysing the levels of inflammatory markers.

Case reports, reviews, letters, and studies which did not present their results in a correct and/or explicit manner, animal studies, and studies where the control groups were made up of patients with other chronic inflammatory disorders were excluded from our meta-analysis. We did not define any minimum number of patients to include a study in our meta-analysis.

### 2.3. Data Extraction

Information was carefully extracted from all eligible publications independently by two authors according to the inclusion and exclusion criteria listed above. Disagreement was resolved by discussion between the two authors. The following data were collected from each study: first author's surname, year of publication, study design, type of cases, type of controls, total numbers of cases and controls, prevalence of traditional risk factors for CVD (obesity, diabetes, smoking, dyslipidemia, and hypertension), the prevalence of* C. pneumoniae* antibodies (IgA or IgG), determined by microimmunofluorescence or ELISA, and the levels of inflammatory markers (CRP, IL-6, fibrinogen, IFN-*γ*, TNF-*α*, ICAM-1, and VCAM-1).

Only inflammatory markers analysed by at least two studies were included in our meta-analysis.

### 2.4. Statistical Analysis

Differences between groups were assessed by means of differences in averages and standard deviations of the difference, as markers assessed were all continuous. Standard errors were computed after a normal approximation. Pooled effects were computed by means of a meta-analysis. The meta-analysis conducted was strictly under heterogeneity among studies, by means of a hierarchical Bayesian model. Effect sizes were assumed to be normally distributed. Each study effect was assumed to arise from a Gaussian centered on a study-specific effect and the extracted standard error, inflated by 25% to obtain a conservative statement. The study-specific effect was assumed to be Gaussian, centered on an unknown pooled effect, which is the main object of interest. An informative prior was used for the variance of the pooled effect, as an inverse Gamma centered on an estimator obtained with a moment-based approach (inflated by 25% to obtain a conservative statement). We also estimated posterior probabilities of no difference (*P*). *P* of less than 0.05 was considered as statistically significant. Potential publication bias was estimated using Egger's linear regression test and funnel plots. Sensitivity analyses were assessed by deleting each study; in all cases, pooled estimates were very stable.

All data were collected using Microsoft Office Excel 2007; statistical analyses were performed using R software.

## 3. Results

### 3.1. Study Characteristics

25 studies concerning the association of* C. pneumoniae* and atherosclerosis through inflammation met the inclusion criteria and comprised 12 case-control, 6 cross-sectional, and 7 prospective studies [44–68]. Overall, a total of 3633 cases and 2781 controls for the case-control analysis, 1593 seropositive and 915 seronegative patients for the* C. pneumoniae* IgG seropositive-seronegative analysis, and 528 seropositive and 726 seronegative patients for the* C. pneumoniae* IgA seropositive-seronegative analysis were considered. One publication had apparently overlapping cases with a second study, so that we extracted only the most relevant data from both studies.  Table 1 presents the main characteristics of the included studies for the case-control analysis, whereas Table 2 presents the main characteristics of the included studies for the seropositive to seronegative analysis according to* C. pneumoniae* IgG and IgA. Controls were mainly healthy population and matched for age and sex.

### 3.2. Meta-Analysis Results

**Figure 1:**
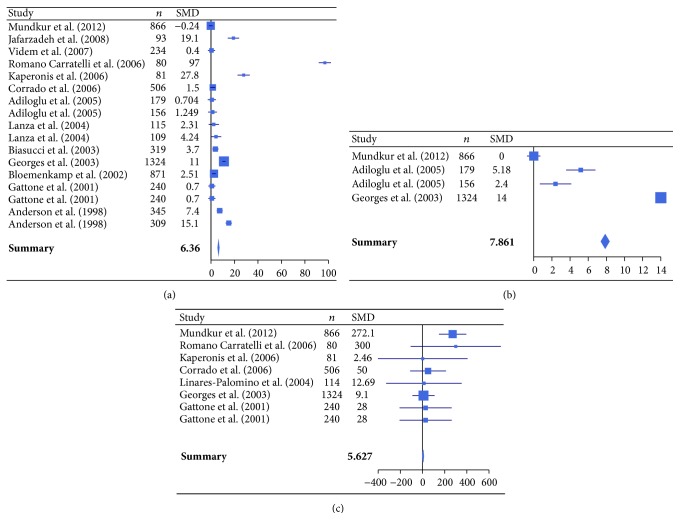
Forest plot of standardized mean differences (SMDs) of individual studies and pooled SMDs for hsCRP (a), IL-6 (b), and fibrinogen (c) in patients with atherosclerotic cardiovascular diseases and healthy controls.

The meta-analysis was performed on 16 studies (case-controls analysis, Table 1) comparing atherosclerotic patients with healthy controls in relation to the seropositivity to* C. pneumoniae* and levels of inflammatory markers (hsCRP, IL-6, fibrinogen, TNF-*α*, IFN-*γ*, ICAM-1, and VCAM-1) [44, 46, 49, 51, 53, 55–57, 59, 61–65, 67, 68].* C. pneumoniae* seroprevalence was 59.2% (95% CI: 57.8–60.7) in cases and 31.2% (95% CI: 30.0–32.5) in controls, with an OR of 3.11 (95% CI: 2.88–3.36, *P* < 0.001). Concerning the inflammatory markers, hsCRP, IL-6, fibrinogen, TNF-*α*, and IFN-*γ* showed a significant increase in patients with atherosclerosis compared to health controls (*P* < 0.05), whereas the adhesion molecules ICAM-1 and VCAM-1 did not show any significant difference (*P* > 0.05) (Table 3, Figure 1).

In addition, the meta-analysis was also performed on studies comparing atherosclerotic patients seropositive to* C. pneumoniae* IgG or IgA with seronegative patients. Specifically, 7 studies comparing atherosclerotic patients seropositive to* C. pneumoniae* IgG with seronegative patients (*C. pneumoniae* IgG analysis, Table 2) [47, 48, 50, 54, 57, 60, 66] and 6 studies comparing atherosclerotic patients seropositive to* C. pneumoniae* IgA with seronegative patients (*C. pneumoniae* IgA analysis, Table 2) [45, 52, 54, 58, 60, 65], both according to levels of inflammatory markers (hsCRP, IL-6, fibrinogen, and IFN-*γ*), were evaluated.

**Figure 2:**
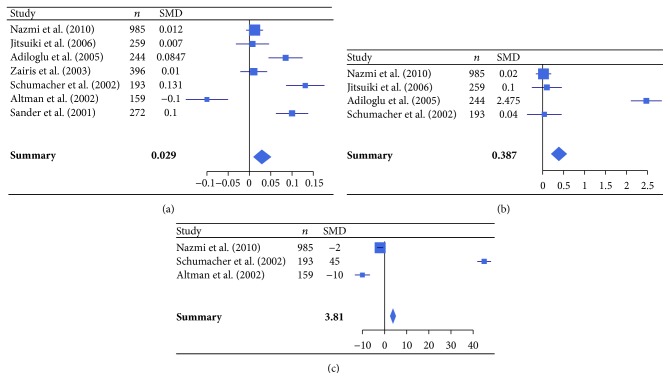
Forest plot of standardized mean differences (SMDs) of individual studies and pooled SMDs for hsCRP (a), IL-6 (b), and fibrinogen (c) in* C. pneumoniae* IgG seropositive and seronegative patients with atherosclerotic cardiovascular diseases.

For the* C. pneumoniae* IgG analysis, hsCRP, IL-6, and fibrinogen levels were significantly higher in seropositive compared to seronegative patients (*P* < 0.0001) (Figure 2).

**Figure 3:**
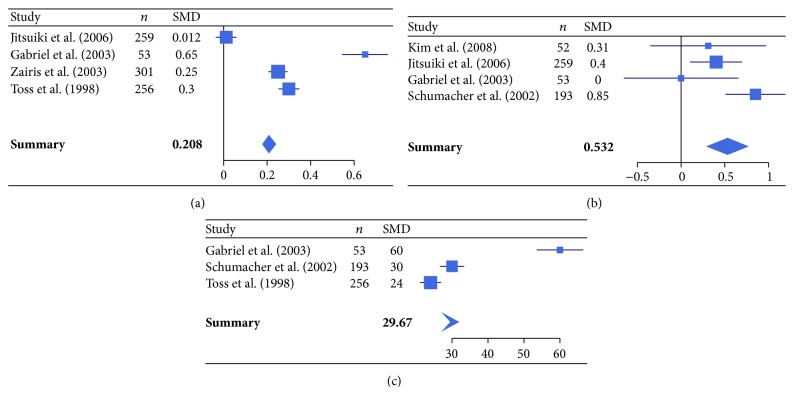
Forest plot of standardized mean differences (SMDs) of individual studies and pooled SMDs for hsCRP (a), IL-6 (b), and fibrinogen (c) in* C. pneumoniae* IgA seropositive and seronegative patients with atherosclerotic cardiovascular diseases.

For the* C. pneumoniae* IgA analysis, hsCRP, IL-6, and fibrinogen were markedly increased in seropositive patients (*P* < 0.01), whereas IFN-*γ* did not show any significant difference between seropositive and seronegative patients (*P* > 0.05) (Figure 3).

Results are independent of the main confounding factors as the meta-analysis was computed under heterogeneity. We also have verified that they could not form a relationship with mean age and study proportions of males, patients with diabetes, hypertension, obesity, smoking, dyslipidemia, and risk factors for CVDs.

### 3.3. Publication Bias

Funnel plots and Egger's test were performed to assess publication bias. Publication bias was detected only for meta-analysis of fibrinogen with IgA (*P* = 0.012). In particular no publication bias was detected for hsCRP for IgA (*P* = 0.200), IgG (*P* = 0.890), and case-control data (*P* = 0.122).

## 4. Discussion

Atherosclerosis, precursor to cardiovascular diseases, is recognised as a chronic inflammatory disease of large arteries, involving cytokines, such as IFN-*γ* and TNF-*α*, adhesion molecules, such as ICAM-1 and VCAM-1, and several plasma inflammatory markers, such as hsCRP, IL-6, and fibrinogen [2, 3]. The latter have been demonstrated to better correlate with the chronic inflammation underlying atherosclerotic cardiovascular diseases; in fact, IL-6, a proinflammatory cytokine that plays a role in the instability of a vulnerable plaque, and hsCRP, an acute-phase protein, appear to be strong independent predictors of cardiovascular events [3]. Lastly, fibrinogen, a substrate leading to the generation of thrombin, the end point of the hemostatic process, is involved in the early formation and growth of the atherosclerotic plaque [69].

As previously described,* C. pneumoniae* has been suggested to contribute to the chronic inflammation underlying the atherosclerotic process, as evidenced by elevated levels of circulating inflammatory markers in atherosclerotic patients. Gattone et al., in 2001, have suggested, for the first time, the association of* C. pneumoniae* seropositivity and high hsCRP levels, with increased risk of myocardial infarction [46]. Since then, other studies [47, 49, 50, 52, 57, 61, 64, 67] have supported the relationship between* C. pneumoniae* infection and atherogenesis through systemic inflammation. On the other hand, some studies have failed to demonstrate such association [44, 48, 55, 68], thus making it difficult to draw conclusions.

To the best of our knowledge, our study is the first meta-analysis to evaluate whether* C. pneumoniae* may contribute to the pathogenesis of atherosclerotic cardiovascular diseases by the means of enhanced inflammation, assessed by levels of inflammatory markers.

The first result of our meta-analysis showed a significant increase in hsCRP, fibrinogen, IL-6, TNF-*α*, and IFN-*γ* in atherosclerotic patients compared to healthy controls. These data, together with the higher seroprevalence of* C. pneumoniae* in atherosclerotic patients compared to healthy controls (*P* < 0.001), suggest that* C. pneumoniae* infection may contribute to the chronic inflammation underlying the development and progression of atherosclerosis, even though it is not possible to establish a causal relationship.

Consequently, in order to better define the contribution of* C. pneumoniae* on the inflammation underlying atherosclerosis, we performed a further analysis on studies comparing the levels of inflammatory markers in atherosclerotic patients seropositive to* C. pneumoniae* IgG or IgA with seronegative patients.

The more relevant data is that* C. pneumoniae* IgA seropositivity was overall more strongly related to the inflammatory state than IgG seropositivity. Indeed, our results showed an increase of inflammatory marker levels, such as hsCRP, fibrinogen, and IL-6, in IgA seropositive patients. Specifically, the standard mean differences of hsCRP, fibrinogen, and IL-6 were, respectively, 7.17, 7.79, and 1.37 times higher in the IgA compared to the IgG analysis, thus showing that seropositivity to* C. pneumoniae* IgA might better correlate to a chronic inflammatory state in patients with atherosclerosis. This is not particularly surprising since* C. pneumoniae* IgA is considered a marker of chronic infection, known to be involved in chronic disorders.

The main strength of our meta-analysis is the statistical robustness of our data, since the results were not affected by exclusion of any specific study from the pooled analysis.

However, our meta-analysis has potential limitations. First, since multiple infectious agents, labeled as infectious burden, rather than any single pathogen, have been shown to contribute to the pathogenesis of atherosclerosis, the inflammatory effect of* C. pneumoniae* might be overestimated. Of note, in all the studies included in our meta-analysis, data regarding other infectious agents were missing or incomplete and, hence, we were not able to quantify their influence on chronic inflammation. Second, potential bias might be introduced in that not all the included studies reported data concerning the confounding factors. Lastly, two different diagnostic tests, along with different cutoffs, were used for detecting* C. pneumoniae* seropositivity in the included studies and, hence, may partly account for further bias.

## 5. Conclusions

In conclusion, despite some limitations, our study suggests that* C. pneumoniae* infection may contribute to atherosclerotic cardiovascular diseases by enhancing the inflammatory state, as demonstrated by increased levels of systemic inflammatory markers. Furthermore, seropositivity to* C. pneumoniae* IgA, together with hsCRP, fibrinogen, and IL-6, may be predictive of atherosclerotic cardiovascular risk.

In the future, large scale, prospective, and well-designed studies should be needed to deepen our knowledge concerning the causative role of* C. pneumoniae* in inflammation in atherosclerotic cardiovascular diseases.

## Supplementary Material

The standard mean differences with 95% confidence interval of inflammatory marker levels, between patients seropositive and seronegative to anti-chlamydial antibodies were assessed. The association of hsCRP, IL-6 and fibrinogen with *C. pneumoniae* IgG (Table S1) or IgA (Table S2) was observed (*P* < 0.0001).

## Figures and Tables

**Table 1:** Characteristics of the studies included in the case-control analysis.

First author	Year	Country	Cases	Number of cases	Number of controls	Study design	Serology assay
Mundkur [68]	2012	India	CAD	433	433	Prospective	ELISA
Haider [67]	2011	India	CAD	63	40	Cross-sectional	ELISA
Jha [65]	2009	India	CAD	192	192	Case-control	ELISA
Jafarzadeh [64]	2008	Iran	IHD	62	31	Case-control	ELISA
Videm [63]	2007	Norway	CAD	131	103	Case-control	ELISA
Corrado [59]	2006	Italy	A	456	212	Prospective	ELISA
Kaperonis [61]	2006	Greece	PAD	51	30	Cross-sectional	ELISA
Romano Carratelli [62]	2006	Italy	CAD	60	20	Case-control	MIF
Adiloglu [57]	2005	Turkey	A	88	91	Case-control	ELISA
Lanza [55]	2004	Italy	SA, cardiac SX	104	60	Case-control	MIF
Linares-Palomino [56]	2004	Spain	PAD	64	50	Case-control	MIF
Biasucci [51]	2003	Italy	UA, MI	259	100	Prospective	MIF
Georges [53]	2003	Germany	SA, UA	991	333	Case-control	MIF
Bloemenkamp [49]	2002	Netherlands	PAD	228	643	Case-control	ELISA
Gattone [46]	2001	Italy	MI	120	120	Case-control	MIF
Anderson [44]	1998	USA	CAD, MI	331	323	Case-control	MIF

CAD, coronary artery disease; IHD, ischemic heart disease; A, atherosclerosis; PAD, peripheral artery disease; SA, stable angina; SX, syndrome X; UA, unstable angina; MI, myocardial infarction.

**Table 2:** Characteristics of the studies included in the *C. pneumoniae* IgG and IgA analysis.

First author	Year	Country	Cases	Number of cases	Number of controls	Study design	Serology assay
IgG							
Nazmi [66]	2010	USA	CAD	697	288	Cross-sectional	MIF
Jitsuiki [60]	2006	Japan	A	136	123	Cross-sectional	ELISA
Adiloglu [57]	2005	Turkey	A	227	17	Case-control	ELISA
Zairis [54]	2003	Greece	SA, UA	182	214	Prospective	MIF
Altman [48]	2002	Argentina	CAD	107	52	Case-control	MIF
Schumacher [50]	2002	Norway	CAD	119	74	Cross-sectional	ELISA
Sander [47]	2001	Germany	TIA, IS	125	147	Prospective	MIF

IgA							
Jha [65]	2009	India	CAD	155	37	Case-control	ELISA
Jitsuiki [60]	2006	Japan	A	92	167	Cross-sectional	ELISA
Schumacher [58]	2005	Norway	CAD	63	130	Cross-sectional	MIF
Gabriel [52]	2003	Sweden	CAD	38	15	Prospective	MIF
Zairis [54]	2003	Greece	SA, UA	87	214	Prospective	MIF
Toss [45]	1998	Sweden	UA	93	163	Prospective	MIF

CAD, coronary artery disease; A, atherosclerosis; SA, stable angina; UA, unstable angina; TIA, transient ischemic attack; IS, ischemic stroke.

**Table 3:** Summary of SMDs and 95% CI of inflammatory marker levels in case-control analysis.

	hsCRP (mg/L)	IL-6 (ng/mL)	Fibrinogen (mg/dL)	TNF-*α* (pg/mL)	IFN-*γ* (pg/mL)	ICAM-1 (ng/mL)	VCAM-1 (ng/mL)
SMD	6.360	7.861	5.627	9.467	4.320	0.001	3.722
95% CI	5.76–6.96	7.40–8.32	0.65–10.60	6.45–12.49	2.32–6.32	−0.25–0.25	−1.46–8.90
*P* value	<0.0001	<0.0001	0.00895	<0.0001	<0.0001	0.49628	0.06611

SMD, standard mean difference; CI, confidence interval.
