# Supplementary material for: Chlamydia pneumoniae-Mediated Inflammation in Atherosclerosis: A Meta-Analysis
Source: Mediators Inflamm. 2015 Aug 9;2015:378658. doi: 10.1155/2015/378658 (PMC4546765; doi:10.1155/2015/378658)
Supplement: Supplementary file 1 — The standard mean differences with 95% confidence interval of inflammatory marker levels, between patients seropositive and seronegative to anti-chlamydial antibodies were assessed. The association of hsCRP, IL-6 and fibrinogen with C. pneumoniae IgG (Table S1) or IgA (Table S2) was observed (P < 0.0001). [file 378658.f1.doc]

| **Table S1: Summary of SMDs and 95% CI of inflammatory marker levels in *C. pneumoniae* IgG analysis.** | | | |
| --- | --- | --- | --- |
|  | hsCRP  (mg/L) | IL-6  (ng/mL) | fibrinogen (mg/dL) |
| SMD | 0.029 | 0.387 | 3.810 |
| 95% CI | 0.01-0.05 | 0.21-0.57 | 2.62-4.998 |
| *P* value | <0.0001 | <0.0001 | <0.0001 |

SMDs, Standard Mean Differences; CI, Confidence Interval.

| **Table S2: Summary of SMDs and 95% CI of inflammatory marker levels in *C. pneumoniae* IgA analysis.** | | | | |
| --- | --- | --- | --- | --- |
|  | hsCRP  (mg/L) | IL-6  (ng/mL) | fibrinogen (mg/dL) | IFN-γ  (pg/mL) |
| SMD | 0.208 | 0.532 | 29.67 | 0.111 |
| 95% CI | 0.18-0.24 | 0.30-0.76 | 27.59-31.75 | -0.07-0.30 |
| *P* value | <0.0001 | 0.00002 | <0.0001 | 0.115 |

SMDs, Standard Mean Differences; CI, Confidence Interval.
